# Flavokawain B, a kava chalcone, inhibits growth of human osteosarcoma cells through G2/M cell cycle arrest and apoptosis

**DOI:** 10.1186/1476-4598-12-55

**Published:** 2013-06-10

**Authors:** Tao Ji, Carol Lin, Lauren S Krill, Ramez Eskander, Yi Guo, Xiaolin Zi, Bang H Hoang

**Affiliations:** 1 Department of Orthopaedic Surgery, UC Irvine Multidisciplinary Sarcoma Center, Chao Family Comprehensive Cancer Center, University of California, Irvine, USA; 2 Musculoskeletal Tumor Center, People’s Hospital, Peking University, Beijing, P. R. China; 3 Department of Oncology, CHOC Children’s Hospital, California, USA; 4 Department of Obstetrics and Gynecology, University of California, Irvine, USA; 5 Department of Urology and Pharmaceutical Science, University of California, Irvine, USA

**Keywords:** Osteosarcoma, Flavokawain, Cell cycle, Apoptosis, Chemotherapeutic, Toxicity

## Abstract

**Background:**

Osteosarcoma (OS) is the most common primary bone malignancy with a high propensity for local invasion and distant metastasis. Limited by the severe toxicity of conventional agents, the therapeutic bottleneck of osteosarcoma still remains unconquered. Flavokawain B (FKB), a kava extract, has been reported to have significant anti-tumor effects on several carcinoma cell lines both *in vitro* and *in vivo*. Its efficacy and low toxicity profile make FKB a promising agent for use as a novel chemotherapeutic agent.

**Results:**

In the current study, we investigated the anti-proliferative and apoptotic effects of FKB against human osteosarcomas. Exposure of OS cells to FKB resulted in apoptosis, evidenced by loss of cell viability, morphological changes and the externalization of phosphatidylserine. Apoptosis induced by FKB resulted in activation of Caspase-3/7, -8 and −9 in OS cell lines, 143B and Saos-2. FKB also down-regulated inhibitory apoptotic markers, including Bcl-2 and Survivin and led to concomitant increases in apoptotic proteins, Bax, Puma and Fas. Therefore, the induction of apoptosis by FKB involved both extrinsic and intrinsic pathways. FKB also caused G2/M phase cell cycle arrest, which was observed through reductions in the levels of cyclin B1, cdc2 and cdc25c and increases in Myt1 levels. Furthermore, migration and invasion ability was decreased by FKB in a dose-dependent manner. The cytotoxicity profile showed FKB had significant lower side effects on bone marrow cells and small intestinal epithelial cells compared with Adriamycin.

**Conclusions:**

Taken together, our evidence of apoptosis and cell cycle arrest by FKB treatment with less toxicity than the standard treatments provides an innovative argument for the use of FKB as a chemotherapeutic and chemopreventive compound. *In vivo* experiments utilizing FKB to reduce tumorigenesis and metastatic potential will be crucial to further justify clinical application.

## Background

Osteosarcoma is the most common primary malignant tumor arising from bone in children and young adults with a very high propensity for local invasion and distant metastasis. Despite new therapeutic developments the survival data for this disease remains unchanged over the past 20 years [1]. Conventional chemotherapy protocols contain a similar three-drug backbone consisting of methotrexate, doxorubicin, and cisplatin (MAP) with the possible inclusion of ifosfamide and etoposide [2]. Given that the current chemotherapy regimens have had limited success in improving metastasis-free survival and the poor response of previously treated patients with relapsed osteosarcoma, we investigated the potential application of natural anticancer agents in treatment of osteosarcoma.

Natural products have played a major role in new drug discovery for centuries, with over 47% of approved anticancer agents being of natural origin [3]. These compounds can be used as antioxidants and cancer preventing agents or cancer therapy drugs. The consumption of kava root extracts in the Pacific Islands has been associated with a lower incidence of cancer [4]. Extracts of kava are classified into two main classes of compounds: kavalactone and chalcone [5,6]. Chalcones have shown anticancer activity via inhibition of cell proliferation, carcinogenesis and metastasis [4,7,8]. The chalcones include Flavokawain A, B and C. Recent studies have shown that flavokawains are apoptotic inducers and anticarcinogenic agents. We, and others, recently demonstrated that flavokawavin B (FKB) induced apoptosis and exhibited both *in vitro* and *in vivo* anticancer activity against bladder, prostate, colon, oral, lung cancer cells, and also mesenchymal tumors including synovial sarcoma and uterine leiomyosarcoma [5,9-14]. These findings encouraged us to investigate the anticancer effects of FKB on OS as a novel compound agent.

## Results

### FKB inhibits proliferation of osteosarcoma cells

**Figure 1:**
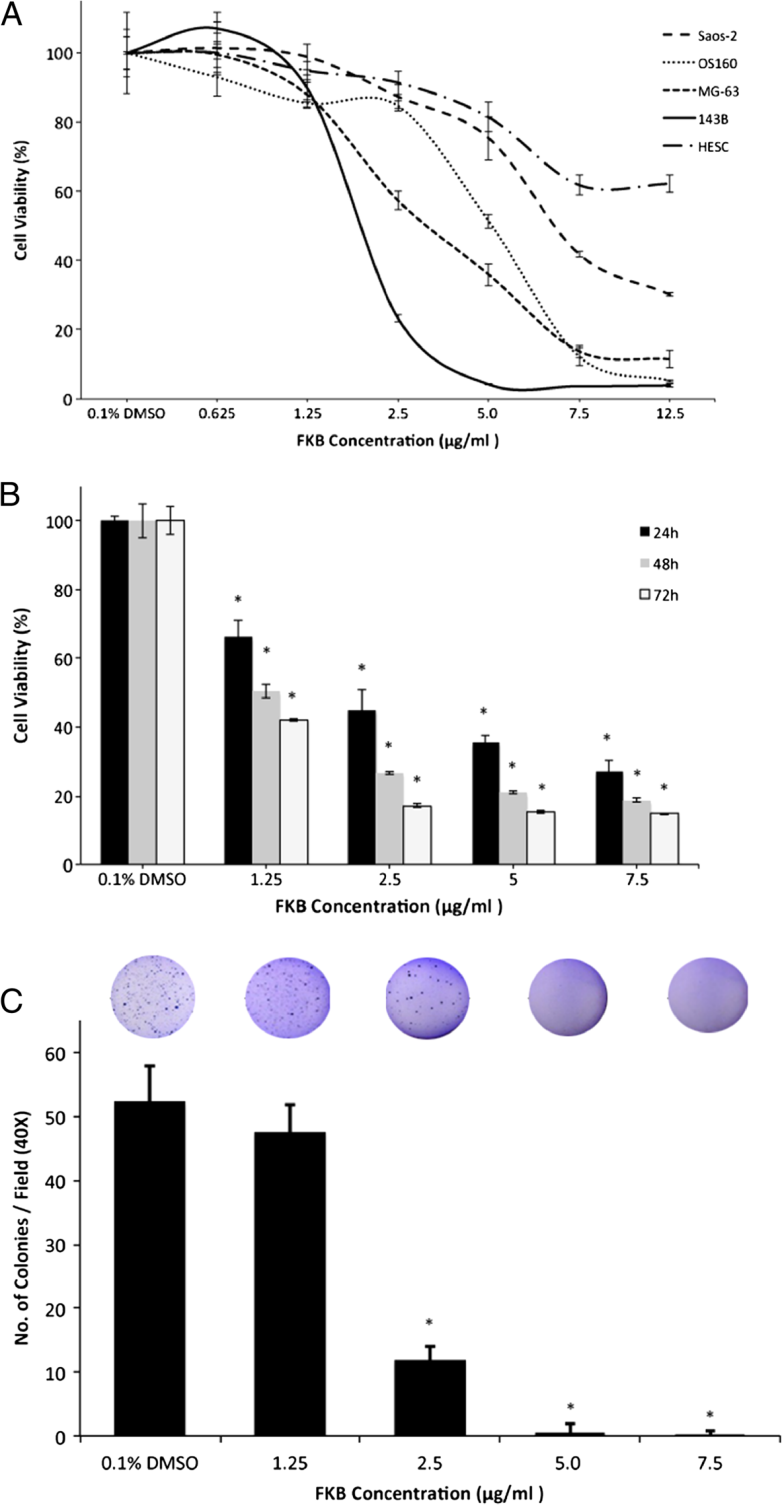
**Antiproliferative effect of FKB on OS cells. A**, Four OS cell lines and fibroblast cell line (HESC) were used and cells were treated with FKB at the indicated concentration in the figure for 72 h, and cell viabilities were measured by MTT assay. **B**, 143B cells were treated with indicated concentrations for 24, 48 or 72 h. **C**, anchorage-independent colony formation assay showed significantly decreased number of colonies formed by 143B cells treated with FKB compared with control group; inset, representative photography of soft agar colonies at 14 days after cell seeding. An asterisk (*) indicates a significant difference in comparison with the control group (p<0.05).

To investigate the effects of FKB on growth, 143B, OS160, MG-63 and Saos-2 cells were exposed to 6 different concentrations for 72 h. Fibroblast cells were used as a control. Figure 1A shows that FKB induced cell death in a dose-dependent manner. FKB at a dose of 5 μg/ml can inhibit the growth of 143B cells by about 90%. The inhibitory effect was also observed in other three osteosarcoma cell lines. The half-inhibitory concentration (IC_50_) of FKB for 72 h on 143B cells was approximately 1.97 μg/ml (3.5 μM). Figure 1B shows that the treatment of 143B cells with FKB resulted in a significant inhibition of cell growth in a time-dependent manner. The 72 h inhibition was more significant than that of 24 h (p<0.05).

The soft agar colony formation assay showed 143B cells formed significantly fewer colonies after FKB treatment (p<0.01, Figure 1C) The results further suggest that treatment of 143B cells with FKB produces result in a significant inhibition of growth in a dose-dependent manner.

### Induction of apoptosis in both 143B and saos-2 cell lines by FKB

**Figure 2:**
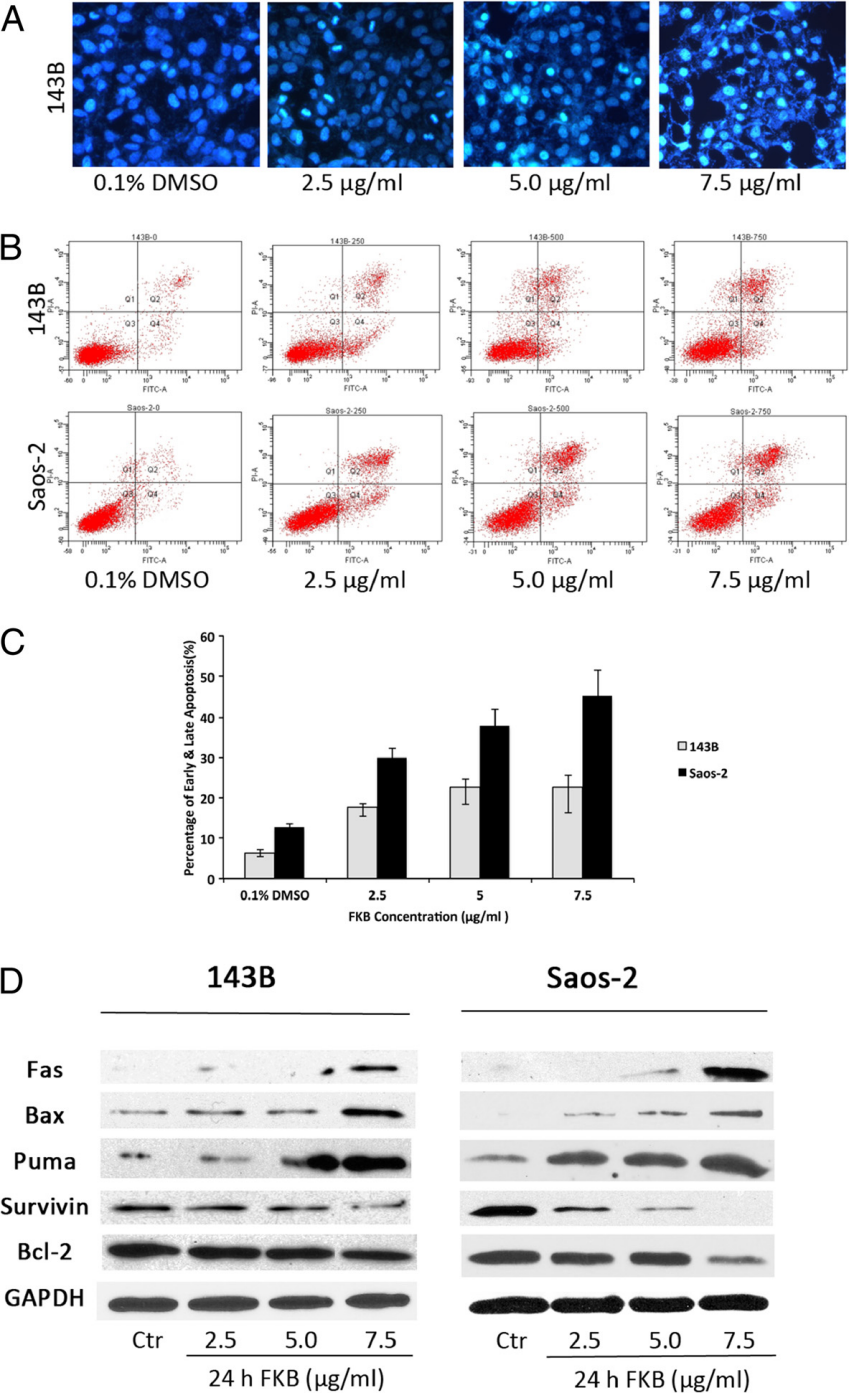
**The apoptotic effect of FKB on OS cells. A**, 143B cells were treated with different concentrations of FKB for 24 h. Apoptosis was evaluated by DAPI staining. **B**, 143B and Saos-2 cells were stained with annexin V and propidium iodide and analyzed by flow-cytometry. **C**, The chart illustrates the results from three separate experiments of flow-cytomety. **D**, FKB treatment induced the expression of Fas, Bax, Puma, and decreased Survivin and Bcl-2 expression. Cells were treated for 24 h and protein was resolved by SDS-PAGE with GAPDH as a control.

To determine whether the inhibition of cell growth by FKB resulted from the induction of apoptosis, morphology study, DAPI staining and FACS were used. The two cell lines exhibited typical apoptotic morphologic changes, including chromatin condensation, separation from surrounding cell, cell shrinkage and cell rounding (data not shown). Following treatment with FKB 24 h, control cells showed round and homogeneous nuclei, whereas cells treated with FKB displayed condensed and fragmented nuclei (Figure 2A). FACS analysis showed that FKB treatment resulted in an increase in both early (lower right) and late apoptotic cells along with the necrotic fractions (upper right) in both 143B and Saos-2 cell lines (Figure 2B and C). The percentage of apoptotic Saos-2 and 143B cells was 45.1±6.4% and 22.7±2.8%, respectively after FKB treatment at the dose of 7.5 μg/ml.

### FKB up-regulates expression of pro-apoptoic protein and down-regulates anti-apototic protein

Apoptosis can be induced via the extrinsic pathway, through cell surface death receptor stimulation, or through the intrinsic pathway mediated by mitochondrial dysfunction [15]. Figure 2D illustrates that FKB treatment of 143B and Saos-2 resulted in increased expression of Fas, Puma and Bax, while down-regulating the expression of Bcl-2 and Survivin. Also, FKB treatment increases Caspase 8, 9, 3/7 activity compared to vehicle-treated controls with a dose-dependent manner (Additional file 1). Taken together, these results imply that FKB activates both extrinsic and intrinsic apoptotic pathways, exhibiting apoptotic effects against osteosarcoma cells.

### FKB suppressed *in vitro* motility and invasiveness

**Figure 3:**
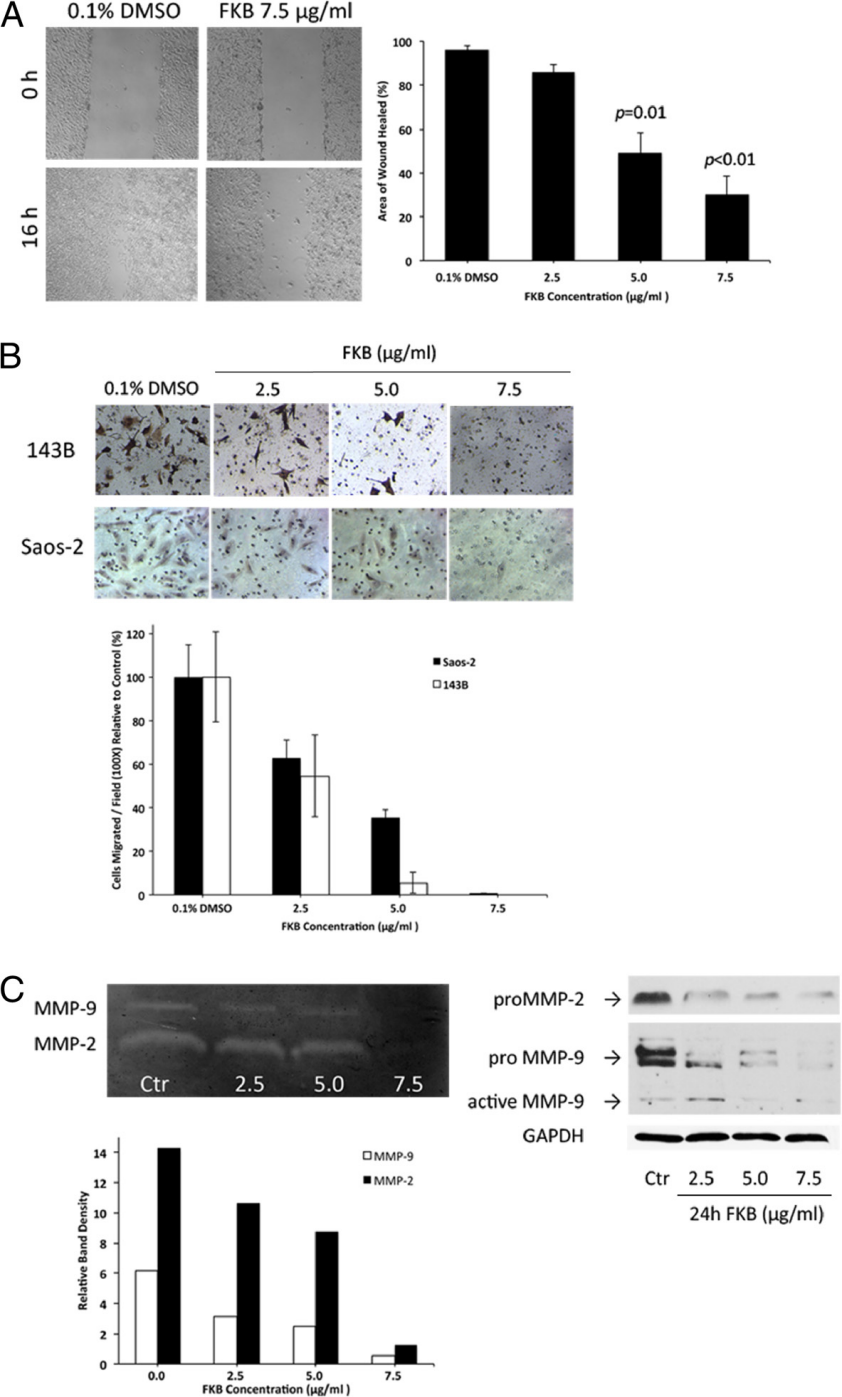
**FKB suppressed cell motility and invasiveness. A**, Representative photomicrographs of scratch wounds were taken at 0 and 16 h after wound were made on 143B treated with FKB 7.5 μg/ml or control. Quantitative measurement of wound healed by ImageJ software showed a reduced cellular motility in FKB-treated 143B cells compared with control group. Columns, mean relative area (%) of wound healed; bars, SD. Experiments were replicated thrice. **B**, Cell invasion ability was analyzed by the Transwell chamber 36 h after FKB treatment at indicated concentration. The number of migrated cells was significantly decreased by FKB in a dose-dependent manner. **C**, Zymography and western blotting analysis of MMP-2 and MMP-9 from cultured medium of 143B cells treated with FKB. Both MMP-2 and MMP-9 activities were significant inhibited by FKB. Also the same inhibition effect of FKB on MMPs was observed on protein expression levels.

To examine whether FKB affect the motility and invasiveness of osteosarcoma cells, we have performed scratch assays. The wound healing area of 143B cells after FKB treatment for 16h was lower than that of control (96.3± 1.8)% with a dose-dependent manner. The migration rate was significantly decreased when the cells were exposed to FKB at the dose of 5.0 μg/ml and 7.5 μg/ml with healed percent of 49.1±9.4 (p=0.01) and 30.1±8.2 (p<0.01), respectively (Figure 3A).

The Matrigel transwell assay showed there was negative correlation between the FKB concentration and the number of osteosarcoma cells that had invaded/migrated through Matrigel. FKB significantly inhibited both 143B and Saos-2 cells invasion in a dose-dependent manner, with 54.6% and 62.7%, respectively (both p=0.01) compared to control at 2.5 μg/ml, 5.5% and 35.4% (p<0.001) at 5.0 μg/ml, and 0% and 0.5% (p<0.001) at 7.5 μg/ml, as shown in Figure 3B.

### Effects of FKB on MMP-2 and MMP-9

Zymography demonstrated MMP-2 and MMP-9 secretion by normal and FKB-treated 143B cells. FKB inhibited the secretion of both MMPs in a dose-dependent manner with almost total inhibition of MMP-9 and MMP-2 at 7.5 μg/ml, as shown in Figure 3C. MMP-2 and MMP-9 secretion level of untreated cells was inhibited by 38.9% and 59.5%, respectively at 5.0 μg/ml FKB and by 91% at 7.5 μg/ml FKB (linear trend, both R^2^=0.93) (Figure 3C). Western blotting showed that FKB reduced the protein levels of MMP-2 and MMP-9.

### FKB induces G2/M arrest in 143B and saos-2 cells

**Figure 4:**
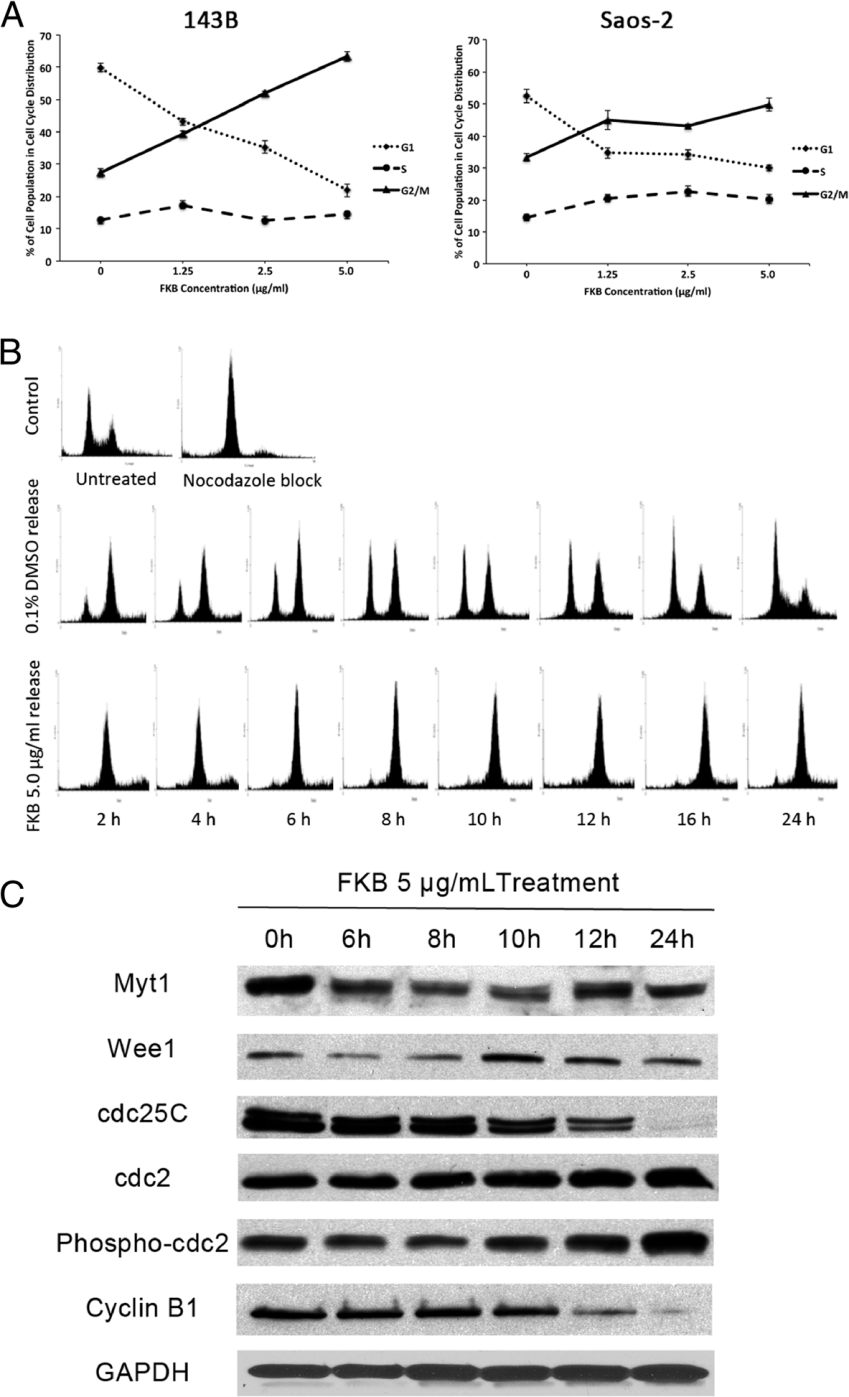
**FKB induced G**_**2**_/**M arrest in OS cells. A**, 143B and Saos-2 cells were treated with vehicle control or FKB at different concentrations for 24 h and analyzed for cell cycle. Percentage of cell cycle distribution after FKB treatment was presented. Points, mean of three samples for each concentration; bars, SE. **B**, 143B cells were blocked at G_2_/M phase and released form the block into either control or 5 μg/ml FKB. At times indicated, cells were collected, fixed, and analyzed by flow cytometry. **C**, The effect of FKB (5 μg/ml) on cell-cycle regulatory markers was examined by Western blot analysis. The protein extracted from 143B cells treated with FKB for indicated time was resolved by SDS-PAGE.

To examine whether FKB treatment could affect cell cycle progression in osteosarcoma cells, asynchronous 143B and Saos-2 cells were treated with different concentrations of FKB. As shown in Figure 4A, FKB treatment results in a marked increase in the number of cells arrested at G2/M phase in both 143B and Saos-2 cell lines in a dose-dependent manner. To further examine the effects of FKB on cell cycle progression we synchronized 143B cells in mitosis phase using nocodazole and subsequently released the cell into FKB 5.0 μg/ml or vehicle control containing media. Analysis of collected cells by flow cytomoetry indicated that control cells progressed normally through mitosis and by 16 hours had lost their synchrony (Figure 4B). In contrast, cells released into FKB (5.0 μg/ml) stayed in M-phase over the time course tested. The cell cycle profile observed was consistent with that previously detected on asynchronous cell lines.

### Effects of FKB on expression of cell-cycle regulator markers

Cell cycle progression is regulated by the cycling actions of the cyclin-CDK complexes and positive and negative regulator proteins [16,17]. Regulation of the cyclin dependent kinase Cdc2 is essential for entry into mitosis. During G2, the Cdc2/Cyclin B complex is kept inactive by phosphorylation of Cdc2 by the kinases Wee1 and Myt1. At the onset of mitosis, both of these residues are dephosphorylated by the phosphatase Cdc25C. Therefore, we hypothesized that the FKB induced G2/M arrest may be caused by inhibition of Cyclin B1, Cdc25C and activation of Wee1 and Myt1. As expected, FKB treatment at 5.0 μg/ml caused significant decrease in Cyclin B1, Cdc 25c and increase in p-Cdc2 in a time-dependent manner (Figure 4C). However, Myt1 showed an increase but not time dependent. No significant increase was found for Wee1 expression. These results imply that FKB inhibit cell cycle progression, at least partially, by decreasing the levels of cdc2, Cyclin B1 and increasing levels of Myt 1 in 143B cells.

### *In vitro* toxicity assay of FKB

**Figure 5:**
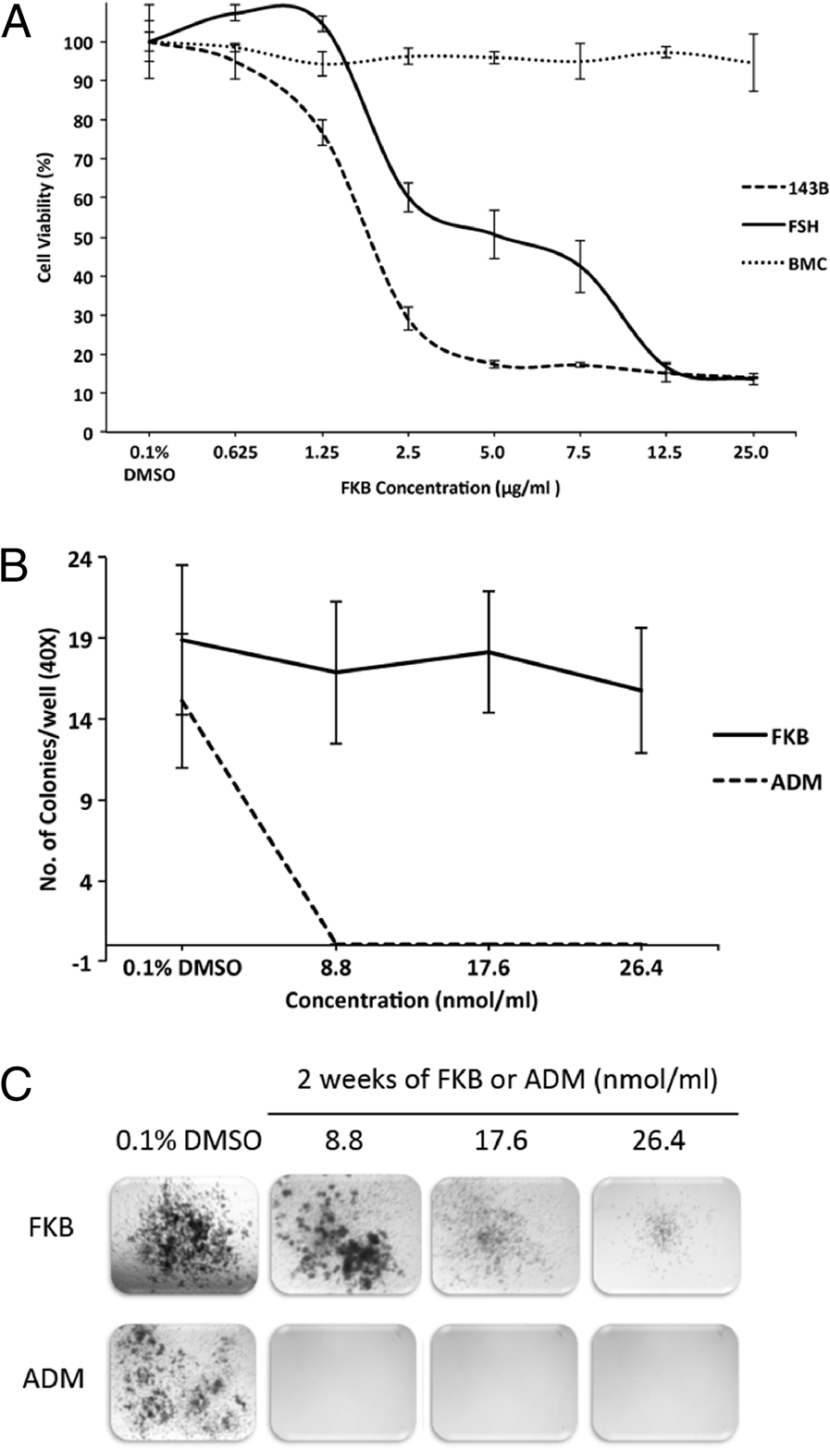
**FKB showed preferential inhibition on 143B cells compared with FHS and bone marrow stem cells. A**, FKB treatment exhibited differential effects on 143B OS cells, normal small intestinal epithelial cell (FHS) and bone marrow stem cells. Cells were treated with FKB for 72 h. **B**, Compared with Adriamycin, FKB showed significantly less bone marrow inhibition effect. **C**, Representative microphotography, taken at 14 days after treatment of bone marrow stem cell colonies showed the size of a typical colony decreased with a higher concentration of FKB.

No significant growth inhibitory effects were observed in the growth of bone marrow cells. Significant differences in cell viability was noted between normal small intestinal epithelial cells and osteosarcoma cells following FKB treatment (p=0.016) (Figure 5A). Bone marrow cell colony formation showed there was no difference in the number of colonies after FKB treatment, however the typical size of colonies decreased in a dose dependent manner (Figure 5B and C). Significant growth inhibition was noted with Adriamycin treatment at all concentrations.

## Discussion

In spite of aggressive treatment protocols including high-dose chemotherapy and wide surgical resection, the long-term survival of patients with localized disease remains between 60-70% during the last two decades [18]. Although maximal dose escalation of conventional chemotherapy has been utilized, there is still no significant gain in clinical outcome. The use of conventional antitumor drugs, such as doxorubicin and methotrexate, is usually limited due to their systemic toxicity and lack of specificity [19]. Furthermore, no effective standard second-line chemotherapeutic agent has been identified in local or distant relapsed osteosarcoma. Many reports have emphasized that use of dietary bioactive compounds is becoming an alternative, safe, and desirable approach to controlling and treating cancer [20]. Our previous studies have shown that FKB exhibits cytotoxic potency against mesenchymal tumors, including synovial sarcoma and uterine leiomyosarcoma [9,12]. The results presented here confirm that FKB could inhibits proliferation of human osteosarcoma cells *in vitro* via G2/M arrest and leads to a robust induction of apoptosis.

We further evaluated the regulatory mechanism for the apoptotic effect of FKB in osteosarcoma cells. Investigations have shown that apoptosis is controlled by both mitochondrial and membrane death receptor pathways. Previous reported research showed that the mechanisms through which FKB induces apoptosis depend primarily on mitochondrial damage [8,20]. The pro-survival protein Bcl-2, combined with Bax, can regulate apoptosis via homologous and heterogeneous complexes. Bax induces the release of cytochrome c and activates the Bax-initiated mitochondria pathway and the capsese 3-dependent apoptotic pathway. Bcl-2 inhibits the realease of cytochrome c against Bax. The disturbance of Bcl-2/Bax protein ratio has been recognized as a factor contributing to the FKB-induced apoptosis [6,20]. In the present study, the increase in Bax and decrease in Bcl-2 was observed in both OS cell lines. Also activity level of caspase-3 was found to increase incrementally with escalating doses. The extrinsic pathway is initiated by the binding of transmembrane death receptors, including Fas, DR5 and TNFR receptors [15]. Activation of Fas receptor leads to receptor clustering and formation of a death-inducing signaling complex, which results in the activation of procapase-8. Then active caspase-8 can then go on trigger the apoptotic caspase cascade. Fas expression may be triggered by FKB treatment and may account for independent activation of caspase-9. Puma is a critical mediator of p53-dependent and p53-independent apoptosis induced by a wide variety of stimuli, including deregulated oncogene expression, toxins, growth factor/cytokine withdrawal, and infection [21]. It has been suggested that Puma can also sponsor apoptosis by directly activating Bax in some cells [22]. Data from the present study suggests that FKB-induced apoptosis is mediated by both mitochondrial and membrane death receptor pathways.

Many conventional anticancer treatments at least partly damage the DNA of cells without specific selectivity selective for cancer cells. Anticancer insights derived from cell cycle research has given birth to the idea of cell cycle G2 checkpoint abrogation as a cancer specific therapy [23]. Several studies have revealed that FKB induce G2/M arrest [12,20,24]. In current study, significant G2/M arrest by FKB in osteosarcoma cells was confirmed by synchronized cell cycle analysis. Further mechanism was explored. The cell cycle blockade was associated with reduction in Cyclin B1 and Cdc25C and increase in Myt1, and phosphorylation-cdc2. During G2, the Cdc2/Cyclin B complex is kept inactive by phosphorylation by the kinase Myt1 [25]. At the onset of mitosis, both residues are dephosphorylated by Cdc25C [26]. Repression of Cyclin B1 and Cdc2 enforces the G2/M arrest. Inhibitory phosphorylation of Cdc2 is essential for the p53-independent G2 arrest that occurs in response to DNA damage, and is dependent on the protein kinases Atm and Atr. The Cdc2 is inactivated by Atm and Atr through increasing phosphorylation of the residues tyrosine 15, which cause G2 arrest in response to DNA damage [17]. We found that FKB did not change the expression level of p53 (Data not shown). Hence, p53-independent G2 arrest may be the main mechanism in FKB-induced cell cycle block.

Results of motility and invasion assays encouraged the potential use of FKB as a new candidate for anticancer therapy against migration and invasion of osteosarcoma cells. Inhibition of motility and invasion with dose-dependent manner was observed in 143B and Saos-2 cell lines. To further explore the exact expression of FKB-induced inhibition of invasion and migration, we performed a gelatin zymography assay to detect the activities of MMP-2 and MMP-9 in 143B cells. The results showed that FKB notably down-regulated activities and protein levels of MMP-2 and MMP-9 in a dose-dependent manner. One of the major characteristics of cancer cell metastasis is altered adhesion ability between cells and the extracellular matrix which is associated with invasion and migration of tumor cells [27]. MMPs are overexpressed in the metastatic tumor cells and have been shown to be involved in the invasion and metastasis of various tumor cells [28]. High MMP-9 expression was observed in pretreatment osteosarcoma tumor samples and in most metastatic lesions, leading to the speculation that MMP-9 is associated with the micrometastatic behavior of osteosarcoma [29]. It is well-established that inhibitions of MMP enzyme activity are early targets for preventing cancer metastasis [30]. Both MMP-2 and MMP-9 are involved with the invasive metastatic potential of tumor cells. The current resutls clearly showed that FKB inhibited the migration and invasion of 143B and Saos-2 cells *in vitro*, which may account for its inhibitory effect on tumor metastasis. Here we found the protein activity of MMP-2 and MMP-9 [28], which are involved in degradation of extracellular matrix and play vital roles in cancer cell migration and invasion.

Any discussion surrounding novel therapeutics must include concerns regarding untoward side effects. The toxicity is an important feature to be considered when a compound is used for treatment, especially for chemopreventive purposes. In order to investigate the potential toxic effect on the resident normal bone marrow mesenchysmal stem cells, we used murine bone marrow cells to study possible toxicity. Notably, the bone marrow cells were quite significantly less sensitive to the FKB, thereby suggesting a preferential toxicity on tumor cells. Compared with adriamycin, FKB showed a considerably lower toxicity on bone marrow cells in the colony formation assay (Figure 5C). FKB was found to have potent hepatocellular toxin [31]. However, the LD_50_ for the two normal liver cell lines was 5- and 10-fold greater than the IC_50_ identified in the current experiment for osteosarcoma cell lines. Based on the *in vitro* results, FKB showed chemotherapeutic effect on tumor cells with significant less toxic effect on normal cells, suggesting its potential use in chemoprevention of OS. Nonetheless, characterization of *in vivo* toxicities related to FKB is highly warranted.

## Conclusion

FKB, a novel chalcone isolated from kava root extracts, showed a strong *in vitro* activity against osteosarcoma cell lines. This compound inhibited cell proliferation, induced apoptosis and cell cycle arrest. Furthermore, the treatment with FKB, in contrast to conventional chemotherapeutic drugs, showed less toxicity in normal bone marrow cells. This implies that FKB may be used as a chemopreventive agent with respect to inhibition of tumor growth, motility and invasion. These preliminary data of FKB treatment on osteosarcoma cells suggest it may enhance the treatment of osteosarcoma.

## Methods

### Cell lines, compounds, and reagents

OS160 was a gift from Dr. Richard Gorlick (Albert Einstein College of Medicine, Bronx, NY). Human OS cell lines 143B , SaOS-2, MG-63 and U2OS were maintained in MEMα medium supplemented with 10% fetal bovine serum. Human small intestinal cell line FHS (American Type Culture Collection, ATCC) were maintained and Hybri-Care Medium. All cells were cultured at 37°C in a humidified incubator with 5% CO2. Pure FKB was purchased from LKT laboratories, dissolved in dimethyl sulfoxide (DMSO), aliquoted, and stored at −20°C. Primary antibodies for Survivin, Bax, Bcl-2, Bcl-xl, Fas, cdc25c, Myt1, cdc2, P-cdc2, Wee1, CyclinB1, P-Chk1(345), GAPDH and secondary antibodies were purchased from Cell Signaling Technology, and antibodies against MMP-2 and MMP-9 were obtained from Thermo Scientific. Thymidine,3-(4,5-dimethylthiazol-2-yl)-2,5-diphenyltetraz-olium bromide (MTT) was obtained from Sigma.

### MTT assay

Briefly, cells were plated into 24-well plates at a density of 2×10^4^ cells in 500 μl of growth medium 24 h prior to treatment. Following treatment with FKB at different doses for 72 h, 500 μL of MTT solution was added to each well and plates were incubated at 37°C for 3 h. The MTT solution was then extracted and 500 μL of dissolving buffer was added to each well. Cell viability was assessed by measuring absorbance at 570 nm in a microplate reader (Bio-Rad). Dose response curves were then created as a percentage of vehicle treated control cells using Excel software.

### Soft agar colony formation assay

Soft agar colony formation assays were done using 6-well plate. Each well contained 2 ml of 0.8% agar in complete medium as the bottom layer, 1 ml of 0.35% agar in complete medium, 6,000 cells as the feeder layer, and 1 ml complete medium as the top layer. Each well was treated with FKB at varying concentrations. Cultures were maintained under standard culture conditions. The number of colonies was determined with an inverted phase-contrast microscope at ×40 magnification. A group of >10 cells was counted as a colony. The data is shown as mean number of colonies±SEM of four independent wells at 14 days after the start of cell seeding.

### DAPI staining for apoptotic cell nuclei

Apoptotic nuclear changes were assessed by ready-to-use DAPI kit (NucBlue™ Fixed Cell Stain, Invitrogen). The cells were seeded into 6-well plates. After treatment with different concentrations of FKB for 24 h, the cells were fixed with Image-IT™ Fix-Perm kit (Invitrogen) as detailed in the package insert. Fluorescence microscopy (Nikon, TE2000-S) was used to observe the apoptotic characteristics of nuclear condensation.

### Caspase activity assay

Apoptosis was confirmed using the Caspase-Glo® 3/7, Caspase-Glo® 8, and Caspase-Glo® 9 Assay (Promega) according to the manufacturer’s instructions. Cells were plated in a 96-well plate and treated with 0.1% DMSO or FKB for 24 h. Then 100 μl reagent were added to each well and the luminescence of each sample was measured in a luminometer (GloMaxVR-MultiDetection System).

### Fluorescence-activated cell sorting (FACS) analysis

FACS analysis of apoptosis was performed utilizing the Annexin V-FITC Apoptosis Detection Kit I (BD Pharmingen) as previously reported [12]. Briefly, 2×10^5^ 143B and Saos-2 cells were seeded into 60-mm dishes 24 h before treatment. Cells were then treated with 0.1% DMSO or different concentrations of FKB for 24 h. Following treatment, the cells were washed with cold phosphate buffered saline (PBS) × 2, and stained with FITC annexin-V/propidium iodide (PI) solution at room temperature, in the dark, for 15 min. Treated samples were then analyzed immediately in a FACSAria flow cytometer (BD Biosciences). The percentage of cells undergoing apoptosis was determined using Multicycle (Phoenix, USA). Annexin-V-FITC(−)/PI(−) was used to indicate cells that had survived, Annexin-V-FITC(+)/PI(−) was used to indicate cells that were in the early stage of apoptosis, and Annexin-V-FITC(+)/PI(+) was used to indicate cells in the late stages of apoptosis or necrosis.

### FACS analysis of cell cycle

Once 143B cells achieved a 70% to 80% confluency, they were treated with 0.1% DMSO or different concentration of FKB for 24 h, or treated with FKB at 5 μg/ml for 2, 4, 6, 8, 10, 12, 16 and 24 h. For synchronization experiment, 143B cells were treated with 100 ng/mL of nocodazole (Sigma) for 24 h at 37°C prior to being released into 0.1% DMSO or FKB at 5 μg/ml. After treatment, cells were fixed in ice-cold 70% ethanol overnight. After fixation, cells were washed thrice with cold PBS and then stained in 500 μl of propidium iodide solution. Samples were analyzed on a BD FACScan flow cytometer and the percentage of cells in the S, G0-G1, and G2-M phases of the cell cycle was determined using WinMDI 2.8.

### Protein isolation and western blot analysis

Samples (normalized according to cell number) were treated with FKB at varying concentrations over 24 h or treated with FKB at 5 μg/μl over different time points. Cell extracts were then prepared in RIPA lysis buffer containing protease inhibitors (Sigma). Cell lysates were centrifuged at 13,000 g for 30 min and the supernatant was collected. The BCA assay was used to determine protein concentration. Volumes of clarified protein lysate containing equal amounts of protein (50 μg) were then separated on 8-12% sodium deodecyl sulfate-polyacrylamide gel electrophoresis (SDS-PAGE) and electrophoretically (90 min at 100 V) transferred to a Hybond-ECL membrane (GE Healthcare). Blots were then blocked for 1 h in TBST containing 5% blocking grade non-fat dry milk (Bio-Rad), and then incubated overnight with primary antibody at 4°C. Blots were then washed three times in TBST and incubated for 1.5 h at room temperature with HRP-conjugated secondary antibody. Immunoreactive bands were visualized using an enhanced chemiluminescence detection system (Thermo Scientific).

### Zymogram assay

To determine proenzyme and active form of MMP-2 and MMP-9, zymogram assay was performed as previously described [32]. In brief, the FBS-free medium was collected from 143B cells treated with FKB or 0.1% DMSO for 72 h, and concentrated using Centricon® Plus-70, 30K NMWL (Millipore). Then the medium with same amount of protein (30 μg) was separated by electrophoresis in 0.1% gelatin-impregnated gel (Bio-Rad). After being re-natured at room temperature for one hour in zymogram re-nature buffer, the gel was incubated overnight at 37°C in zymogram development buffer (Bio-Rad). Gel was then stained with SimplyBlue SafeStain (Invitrogen) and destained according to the manufacturer’s protocols (Bio-Rad). Gelatinolytic activity was visualized as clear bands on the gel. The gel was then scanned and relative changes in bands were measured by densitometry using ImageJ software.

### Motility and invasion assay

Motility was assessed with a scratch assay [33]. Cells were seeded in a 6-well plate at a density of 1×10^6^ cells/well in growth medium until they reached a confluence of 90% approximately. A scratch was made through each well using a sterile pipette tip. The monolayer was incubated with a migration assay buffer consisting of serum-free medium and different concentration of FKB. Images were captured at the same position at 0 h and 16 h. The area of wound healed was calculated with ImageJ software [34]. Invasion assay were performed using 24-well invasion chamber system (BD Biosciences). The matrigel coated inserts were used for invasion assay. 1×10^5^ cells were seeded in the upper chamber in serum-free MEMα medium. MEMα medium with 10% FBS was placed in the bottom well. Incubation was carried out for 36 h at 37°C in humidified air with 5% CO_2_. Non-invaded cells in the upper chamber were then removed with a cotton swab. Invaded cells beneath the bottom membrane of the inserts were fixed with methanol and stained with hematoxylin. The number of invading or migrating cells was determined by counting five fields (100×) under the microscope, and calculated as mean number of cells per field. All the research was performed in triplicate.

### *In vitro* cytotoxicity assay

FHS cell line (ATCC) and murine bone marrow cells (derived from Balb/c, 6–8 weeks old) were used in cytotoxicity assays. The isolation of bone marrow stem cells was performed according to previously reported methods [35]. The animal protocol was approved by the Institutional Animal Care Utilization Committee. FKB toxicity to bone marrow stem cells and small intestinal epithelial cells was tested by the cell counting kit-8 (Kumamoto, Japan) after being exposed to different concentrations of FKB for 72 h and measured by microplate reader scanning at 450 nm as described elsewhere [36]. Also the 143B cells were used as control. Colony formation by mice bone marrow cells was used to investigate the possible inhibitory effect of FKB on bone marrow cells. After the bone marrow cells were isolated, the yield and viability of cells was determined by Trypan blue exculsion and counted on a hemocytometer. A total number of 2×10^4^ cells were mixed with FKB or Adriamycin at concentration of 8.8 nM/ml, 17.6 nM/ml and 26.4 nM/ml, respectively. The mixture was cultured in 1 ml ColonyGel™ 1201 Mouse Base Medium (Reachbio) using a 6-well plate under standard culture conditions for 2 weeks. The number of colonies was determined with an inverted phase-contrast microscope at ×40 magnification. A group of >10 cells was counted as a colony.

### Statistical analysis

The data are presented as means ± standard errors (SE). The level of significance was set at a P < 0.05. Comparison of the differences between treated and control groups were performed using the student’s t-test. All statistical tests were two-sided. R^2^ value of correlation was determined for MMP activity correlations to the FKB concentration using Excel mac 2011.

## Competing interests

The authors declare that they have no competing interests.

## Authors’ contributions

BHH and TJ conceived and designed study; analyzed and interpreted data; drafted and revised the manuscript. TJ and CL performed experiments and the statistical analyses. RE and KL assisted with Western Blot and Zymography. ZL assisted with cell cycle analysis. All authors read and approved the manuscript.

## Supplementary Material

Additional file 1 FKB induces apoptosis and activates caspase 3/7, 8, and 9 in 143B cells (*p<0.05). Click here for file
