# Supplementary figures and images for: Flavokawain B, a kava chalcone, inhibits growth of human osteosarcoma cells through G2/M cell cycle arrest and apoptosis
Source: Mol Cancer. 2013 Jun 10;12:55. doi: 10.1186/1476-4598-12-55 (PMC3681603; doi:10.1186/1476-4598-12-55)

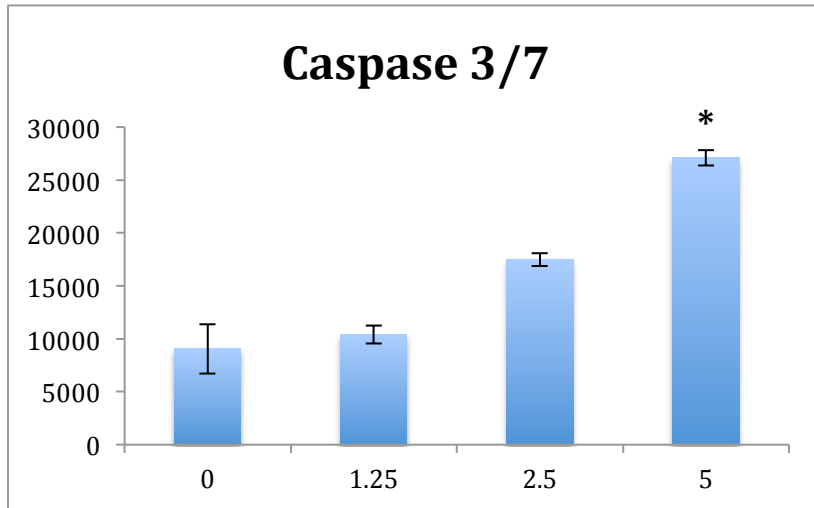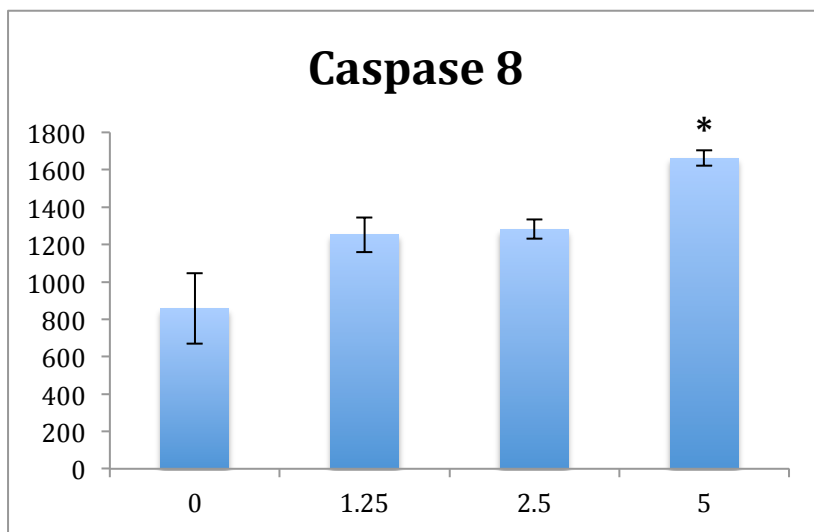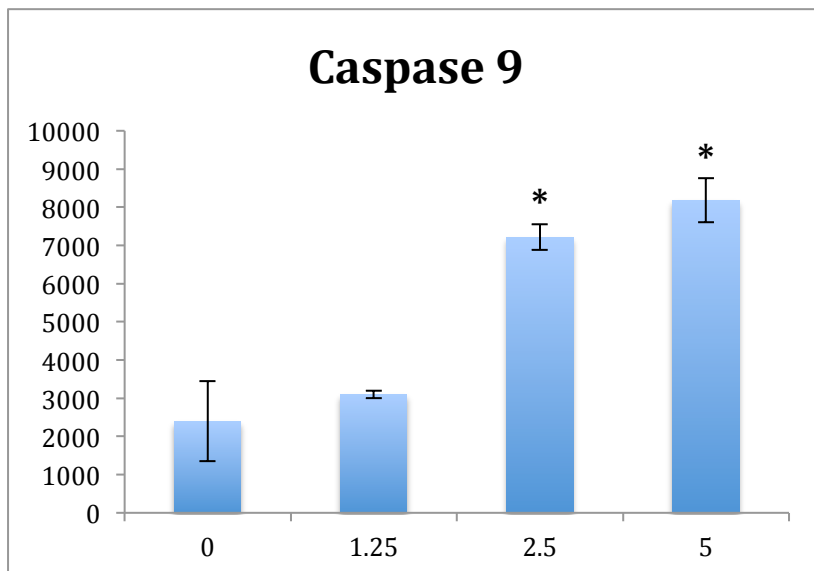

Supplement: Additional file 1 — FKB induces apoptosis and activates caspase 3/7, 8, and 9 in 143B cells (*p<0.05). [file 1476-4598-12-55-S1.pdf]
